# Endometrial polyps—neoplastic lesions or not? Is it time to close the files?

**DOI:** 10.1038/s41379-022-01162-z

**Published:** 2022-09-24

**Authors:** Jörn Bullerdiek, Burkhard M. Helmke, Mohamed Laban

**Affiliations:** 1 Institute of Medical Genetics, University Rostock Medical Center, Rostock, Germany; 2 Institute of Pathology, Elbe Kliniken, Klinikum Stade, Stade, Germany; 3 Gynecologic Oncology Unit, Ain Shams University Hospitals, Cairo, Egypt

## To the Editor:

Endometrial polyps (EMPs) are benign lesions with disorganized proliferation of endometrial glands histologically displaying irregularly shaped glands, hypercellular, hypocellular, or fibrous stroma as well as thick-walled blood vessels.

In a recent interesting contribution to this journal Sahoo et al. (2022)^1^ have addressed the question of driver mutations that may give rise to neoplastic transformation of these frequent histologic lesions. In their study, a combined approach was carried out in EMPs from 31 women using exon sequencing of a large panel of tumor-related genes including oncogenes, tumor suppressors, and chromosomal translocation partners and applying fluorescence in situ hybridization (FISH) with break-apart probes for *HMGA1* and *HMGA2* in a subset of 16 of these lesions. Previous studies mostly published in the early 1990s have identified recurrent translocations or inversions of chromosome bands 12q14-14 or 6p21 in a considerable percentage of EMPs (see e.g.^2–7^.). As to the translocations there is a clear similarity with those observed in uterine fibroids with some differences. Most remarkably, the t(6;14)(p21;q24) is much more frequent in EMPs than in fibroids^7^. In the middle of the 1990s it was shown that the molecular basis of translocations involving 12q14-14 or 6p21 are rearrangements affecting either of the two gene loci of the two high mobility protein genes *HMGA2* or *HMGA1*, respectively, corresponding to their overexpression which can also detected by immunohistochemistry^8–10^. Some of these latter studies showed that these rearrangements are confined to the stromal part of EMPs suggesting that they represent truly benign neoplasms driven by the corresponding genetic rearrangements of either *HMGA1* or *HMGA2*.

The recent study by Sahoo and coworkers, however, is challenging this interpretation because the results failed to detect *HMGA*-gene rearrangements in the lesions analyzed. They found no evidence for any recurrent chromosomal rearrangements, and also by copy-number analyses no evidence of significant chromosome-level events at all was obtained. As an explanation Sahoo et al. propose that a limitation of classical cytogenetics through karyotyping is the occurrence of chromosomal rearrangements that represent tissue culture artifacts. Also, they state that FISH to independently validate these rearrangements was not available in the 1990s. Unfortunately, neither of these statements can explain the early finding of these rearrangements or their lack in the study by Sahoo et al. (2022).

First, there is no evidence that *HMGA*-gene rearrangements seen in a variety of benign tumors may represent artifacts caused caused by cell culture procedures. This would require an in vitro-origin of the chromosomal aberrations and their rapid clonal spreading across the corresponding cell cultures accompanied by a high selective advantage under these conditions. Secondly, as to the FISH this method was not only well-available in the 1990s but also has been broadly applied to the molecular characterization of a 6p21 and 12q14-14 alterations seen in a couple of human benign tumors including EMPs. It was the basis of the identification of *HMGA1* or *HMGA2* as their target genes^8,9^. Accordingly, the conclusions as proposed by Sahoo et al. are not suited to explain the discrepancies between the early findings of *HMGA*-gene rearrangements and the lack of detection in their recent study. Thus, what else could be a sufficient explanation?

The rearrangements that have been found closely resemble those in uterine fibroids and are apparently confined to the stromal part of the EMPs^5,10^. It seems very unlikely that in all cases usually investigated by experienced pathologists submucosal fibroids were studied instead of EMPs. In addition, the histology of some of the lesions studied by conventional cytogenetics and/or FISH has been shown for some of the positive lesions (see e.g^10^.). For us, a more likely explanation is that exon sequencing as performed by Sahoo et al. (2022) will miss some of the cases with rearrangements because we have to expect a high proportion of lesions with extragenic breakpoints like it is the case e.g. also in uterine fibroids as well as in pulmonary chondroid hamartomas. Also, for the same reason some of the cases may have escaped the detection by FISH though the break-apart probes as used in general seem to be better suited to detect these rearrangements. Finally, yet another explanation for the lack of HMGA-gene rearrangements may be that the study by Sahoo et al. did only include samples of at least 50-year old patients. Nevertheless, a considerable percentage of 30-40% of EMPs occur in younger women. To the best of our knowledge there is no data available excluding a more frequent occurrence of the latter rearrangements in younger women.

However, we do not agree with the conclusion by Sahoo and coworkers that EMPs are non-neoplastic. Instead, the majority of data available still point to a subset of EP that are stroma-derived benign tumors driven by rearrangements of either *HMGA1* or *HMGA2* with no significant risk to undergo malignant transformation towards sarcomas. Nevertheless, the data by Sahoo et al. have shown the occurrence of mutations in their epithelial part which in part overlap with those mutations found in endometrial carcinoma or carcinosarcoma.

In summary, we do not feel that it is time to close the files on the pathogenesis of EMPs. Further investigations on the prevalence of possible driver mutations seem to be necessary. One other approach could be the investigation of a larger number of EMPs by immunohistochemistry for the overexpression of HMGA1 and HMGA2, respectively, followed by an in depth molecular characterization of cases revealing positivity.
